# A Soluble Form of the High Affinity IgE Receptor, Fc-Epsilon-RI, Circulates in Human Serum

**DOI:** 10.1371/journal.pone.0019098

**Published:** 2011-04-22

**Authors:** Eleonora Dehlink, Barbara Platzer, Alexandra H. Baker, Jessica LaRosa, Michael Pardo, Peter Dwyer, Elizabeth H. Yen, Zsolt Szépfalusi, Samuel Nurko, Edda Fiebiger

**Affiliations:** 1 Division of Gastroenterology and Nutrition, Department of Pediatrics, Harvard Medical School, Children's Hospital Boston, Boston, Massachusetts, United States of America; 2 Department of Pediatrics and Adolescent Medicine, Medical University of Vienna, Vienna, Austria; Centre de Recherche Public de la Santé (CRP-Santé), Luxembourg

## Abstract

Soluble IgE receptors are potential *in vivo* modulators of IgE-mediated immune responses and are thus important for our basic understanding of allergic responses. We here characterize a novel soluble version of the IgE-binding alpha-chain of Fc-epsilon-RI (sFcεRI), the high affinity receptor for IgE. sFcεRI immunoprecipitates as a protein of ∼40 kDa and contains an intact IgE-binding site. In human serum, sFcεRI is found as a soluble free IgE receptor as well as a complex with IgE. Using a newly established ELISA, we show that serum sFcεRI levels correlate with serum IgE in patients with elevated IgE. We also show that serum of individuals with normal IgE levels can be found to contain high levels of sFcεRI. After IgE-antigen-mediated crosslinking of surface FcεRI, we detect sFcεRI in the exosome-depleted, soluble fraction of cell culture supernatants. We further show that sFcεRI can block binding of IgE to FcεRI expressed at the cell surface. In summary, we here describe the alpha-chain of FcεRI as a circulating soluble IgE receptor isoform in human serum.

## Introduction

Allergic patients are commonly characterized by high serum IgE and high IgE-receptor expression on effector cells of the innate and adaptive immune system [1], [2]. In humans, three different IgE-receptors have been described: CD23, galectin-3 and FcεRI [1], [2]. CD23, also known as FcεRII, is a low affinity IgE receptor and the classical IgE receptor on B cells. Galectin-3, formerly known as epsilon binding protein (εBP), is another low affinity IgE receptor; its role in allergy is rather poorly defined [3], [4]. FcεRI, the high-affinity receptor for IgE, induces activation of mast cells and basophils via IgE-antigen complexes during the acute phase of an allergic response [5], [6]. In rodents, FcεRI is constitutively expressed on the surface of basophils and mast cells as a tetrameric receptor composed of the ligand-binding alpha-chain, one beta-chain and a pair of disulphide-linked gamma-chains. Humans can express a trimeric version of FcεRI lacking the beta-chain on eosinophils and antigen presenting cells, such as dendritic cells and Langerhans cells [6], [7]. Additionally, expression of FcεRI on bronchial and intestinal epithelial cells was described in humans [8], [9]. Serum IgE binding stabilizes surface FcεRI leading to the upregulation of receptor levels in allergic patients [10], [11], [12].

In addition to the transmembrane forms, CD23 and galectin-3 are found as soluble proteins in human serum. Soluble CD23 (sCD23) is a modulator of IgE responses *in vivo* and is generated by cleavage of membrane CD23 from the surface of B-cells [13]. sCD23 has been demonstrated to enhance IgE production [14], [15], [16] and several reports show that high serum levels of sCD23 correlate directly with the severity of allergy and asthma [17]. Along this line, successful immune therapy is accompanied by a drop in sCD23 levels in the serum of allergic patients [18]. The role of sCD23 in modulating IgE production and its potential for monitoring allergic responses has been discussed for more than two decades [13], [19], [20]. However, sCD23 is currently approved as a prognostic parameter only for B-cell chronic lymphocytic leukemia [21], [22], [23]. Interestingly, soluble galactin-3 is also a common marker for tumor burden [4], [24]. Why the production of these soluble IgE receptors is induced during malignant diseases is an interesting scientific question that has yet to be resolved. Thus, our limited understanding of the *in vivo* role of sCD23 and soluble galectin-3 highlights the need for continued research on soluble factors that modulate serum IgE responses in the context of an allergic response.

FcεRI is an activating immune receptor of the immunoglobulin superfamily, which includes the Fc receptors CD16, CD32, CD64 and CD89 [6], [25], [26]. FcεRI shares key structural characteristics and signaling features with these Fc receptors. For most IgE, IgG and IgA Fc receptors, soluble isoforms are found in humans. FcεRI, however, has so far not been reported as a soluble IgE receptor in human serum [1], [6].

Here we describe a soluble form of the FcεRI alpha-chain (sFcεRI). In human serum, this sFcεRI is found as both a free form and bound to its ligand IgE. We show that IgE-mediated cell activation induces the release of sFcεRI *in vitro* and that the soluble form of the receptor can inhibit binding of IgE to FcεRI at the cell surface.

## Results

### Detection of a soluble form of FcεRI alpha (sFcεRI) in human serum

**Figure 1:**
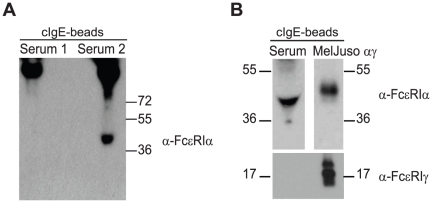
A soluble form of the high affinity IgE receptor, FcεRI, is found in human serum. A. Immunoprecipitations from a negative (first lane, Serum 1) and a positive (right lane, Serum 2) serum specimens. Soluble FcεRI (sFcεRI) was precipitated from serum with IgE-loaded NIP-beads and eluted with non-reducing Laemmli sample buffer. Eluates were separated on 12% non-reducing SDS-PAGE gels, transferred to PVDF membranes and probed with anti-FcεRI-alpha mAb 19-1 followed by peroxidase (HRP)-conjugated goat-anti-mouse IgG for detection of precipitated α-chain. B. Comparison of sFcεRI from serum (upper left blot) with FcεRI precipitated from the cell surface of MelJuso-αγ cells (upper right blot). In the low molecular weight range, blots were re-probed with an anti-FcεRI-gamma polyclonal serum. sFcεRI does not associate with the gamma chain (lower panel). Molecular weight is given in kDa.

To give a definitive answer whether a soluble form of the alpha chain of FcεRI exists in humans, we performed immunoprecipitation experiments to isolate this protein from serum. Sera from patients with normal IgE levels and elevated IgE were run over IgE-columns. Eluates from these columns were analyzed with the FcεRI alpha-chain specific mAb 19-1 by Western blot [12]. The IgE used for these precipitation is commonly used for detection of FcεRI [12], [27] and has a chimeric immunoglobulin containing the human IgE heavy chain and a murine Fab-anti NP fragment (referred to as cIgE from here on). Columns were prepared by coupling cIgE to NP sepharose. 10 ml serum was run over a gravity column packed with 0.5 ml beads. Figure 1A shows a representative positive (right lane) and a negative serum (left lane). A soluble form of FcεRI-alpha (sFcεRI) was precipitated as a protein of ∼40 kDa (Figure 1A). The higher molecular weight bands of the Western blot shown in Figure 1A (≥130 kDa) are a result of cross-reactivity of the secondary anti-mouse antibody used for immunoblotting and the precipitating cIgE. Only the low-molecular weight protein from the serum precipitate is recognized specifically by the anti-FcεRI alpha-chain specific mAb 19-1. Since this antibody recognizes the IgE binding epitope of FcεRI alpha [12], [28], these data show a soluble non-IgE bound form of the receptor in human serum. When the mAb 19-1 was replaced with an isotype control antibody, the sFcεRI band was no longer detected (data not shown). Individuals with normal to moderately elevated IgE levels tested strongest positive in the immunoprecipitation assay. In such sera the sFcεRI is likely still available for precipitation, whereas in patients with elevated IgE the soluble receptor is mainly bound to serum IgE and therefore cannot be immunoprecipated by the mAb19-1.

To perform a more detailed molecular characterization of sFcεRI, we next compared sFcεRI-alpha precipitated from serum to FcεRI-alpha precipitated from the cell surface of MelJuso-αγ cells. As expected for a soluble form, sFcεRI has a lower molecular weight than the surface expressed protein. Unlike transmembrane FcεRI-alpha, which forms a multimeric complex with the common FcR-gamma chain (also called FcεRI-gamma) [1], [6], sFcεRI was not associated with FcεR-gamma (Figure 1B). This finding confirms that sFcεRI is likely a soluble serum protein that is distinct from the membrane multimeric form of the receptor [6], [28].

In summary, this set of results show that the Fc-portion of human IgE can interact with a soluble alpha-chain protein in serum and that this serum sFcεRI does not have the molecular characteristics of the multimeric membrane-associated FcεRI.

### Detection of sFcεRI in human serum by ELISA

**Figure 2:**
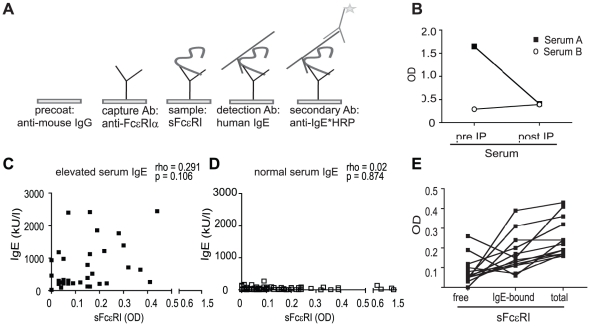
Detection of sFcεRI in human serum by ELISA. A. Schematic of the ELISA established for the detection of sFcεRI. B. ELISA measurements pre- and post-immunoprecipitation with IgE-loaded NIP-beads confirmed that IgE immunoprecipitation depleted serum of sFcεRI. OD, optical density at 450 nm. C. In children with elevated IgE-levels, levels of sFcεRI and total IgE levels correlate. D. In children with normal serum IgE-levels, sFcεRI could be detected, but no correlation with total IgE levels was found. E. sFcεRI circulates as a free or an IgE-complexed protein in human serum. By omitting the IgE-loading step in the ELISA protocol, circulating complexes of IgE and sFcεRI were measured. The fraction of free sFcεRI was then calculated as OD(total sFcεRI)−OD(IgE- sFcεRI complexes) = OD (free sFcεRI). Graph displays the 14 patients with the highest OD (total sFcεRI) with an arbitrary cut off of >0.15.

To allow for semi-quantitative analysis of sFcεRI levels in human serum, we next established a sandwich-ELISA system (schematic model in Figure 2A). For this ELISA, the anti-FcεRI-alpha mAb CRA1 was used as the capture antibody. This mAb binds the stalk region of the alpha-chain and is expected to capture a serum alpha-chain without interfering with the IgE-binding epitope [29]. Levels of serum sFcεRI dropped when comparing pre- and post-immunoprecipitation samples (Figure 2B), confirming the specificity of our ELISA. As a further control, capture and detection antibodies were omitted, which consistently resulted in a loss of sFcεRI signal (data not shown). We were also able to detect sFcεRI in plasma with this ELISA, (data not shown). Using a small collective of atopic pediatric patients (5 boys, 3 girls, mean age 10.3+/−2.7 years; detailed patient characteristics are found in Table S1), we established that this ELISA is a feasible method for detection of sFcεRI in a larger patient set. In summary, this set of data describes a novel ELISA for the detection of serum sFcεRI. Any conclusions about the clinical relevance of this finding are however not possible based on the small patient collective.

### Serum levels of sFcεRI correlate with serum IgE levels in patients with elevated IgE

Due to the absence of a recombinant sFcεRI protein for the generation of a standard curve, the specific, blanked OD was used for semi-quantitative analysis of serum sFcεRI levels. To investigate the occurrence of sFcεRI and potential associations with serum IgE in pediatric patients, we screened sera from a cohort of 119 children (56 boys, 63 girls, mean age 10.6+/−5.3 years) with a wide range of normal and elevated serum IgE levels. Patients were categorized based on the specifications given in the Materials and Methods section into individuals with normal or elevated IgE. We found a weak correlation between serum IgE and sFcεRI in patients with elevated IgE (n = 32, rho = 0.291, p = 0.106, Spearman's rank correlation, Figure 2C). In children with normal IgE levels, sFcεRI was also found, but no correlation to serum IgE was detected (n = 87, rho = 0.02, p = 0.874, Spearman's rank correlation, Figure 2D). Interestingly, patients with highest levels of sFcεRI did not show elevated serum IgE levels (Figure 2D). In an independent experiment, we confirmed that sFcεRI itself did not interfere with detection of IgE and vice versa (data not shown). One limitation of the analyzed patient cohort is that it does not contain healthy individuals, because truly healthy children do not undergo upper GI tract endoscopy for diagnostic purposes. Our patients are controls with regards to the non-inflamed esophageal tissue, but show clinical symptoms of yet unclassified nature. For conclusions on the clinical relevance of our findings it is therefore important to study sFcεRI in sera from truly healthy controls.

### sFcεRI circulates as a free or an IgE-complexed protein in human serum

The alpha-chain of FcεRI has a high-affinity-binding site for IgE [6]. It is thus likely that sFcεRI exists as a preformed complex with IgE in human serum. By omitting the IgE incubation step in our ELISA and detection with the anti-human IgE-HRP conjugate, our method allowed for the detection of sFcεRI-IgE complexes in serum. Subtracting the signal without the *in vitro* IgE incubation step from the signal with the IgE incubation step allowed us to determine how much sFcεRI was complexed to IgE *in vivo*. We randomly selected 14 sera that were positive for sFcεRI and found that in human serum, sFcεRI is present as both a free and an IgE-bound protein (Figure 2E).

### IgE-antigen-mediated receptor crosslinking induces the production of sFcεRI from an FcεRI-expressing cell line

**Figure 3:**
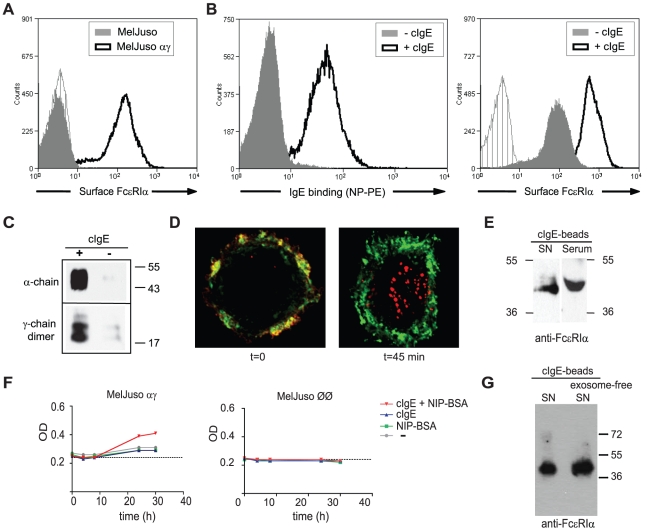
sFcεRI is released from a cell line after IgE-antigen-mediated receptor crosslinking. A. Surface FcεRIα expression of MelJuso-αγ cells (black histogram) compared to MelJuso cells (filled gray histogram) and isotype control (grey hedged histogram). B. Detection of IgE binding of MelJuso-αγ cells with NP-PE (left overlay) and of increased surface FcεRIα expression induced by IgE binding (right overlay). Cells were cultured for 16 h in the presence or absence of cIgE. Black histograms represent cells incubated with cIgE, grey filled histograms are non-cIgE treated cells; isotype control is shown as gray, hedged histogram. C. Coimmunopreciptiation of FcεRI alpha and gamma-chain dimers from MelJuso-αγ cells. D. FcεRI internalization induced by antibody-mediated crosslinking. FcεRI is shown in red and cell surface membranes were stained with WGA (in green). Representative images of cells with non-crosslinked FcεRI (t = 0; left image) and cells 45 min after crosslinking of FcεRI (t = 45 min; right image). E. After 36 h of crosslinking, sFcεRI can be precipitated from supernatants of activated MelJuso-αγ. sFcεRI proteins were precipitated with IgE-loaded -beads, eluted with non-reducing Laemmli sample buffer, separated on 12% non-reducing SDS-PAGE gels, transferred to PVDF membranes, and probed with anti-FcεRI-alpha (mAb 19-1) followed by peroxidase (HRP)-conjugated goat-anti-mouse IgG. F. Kinetics of sFcεRI release into culture supernatants. Supernatants were harvested from MelJuso-αγ cell cultures 4, 8, 24 and 32 h after receptor activation (left graph). ELISA measurements showed an accumulation of sFcεRI over time. MelJusoØØ does not produce sFcεRI (right graph). G. sFcεRI is a soluble protein as it could be detected in culture supernatants (left lane) and in exosome-depleted culture supernatants (right lane). SN: supernatant; OD: optical density. Molecular weight is given in kDa.

The mechanism of sFcεRI production cannot be studied using primary human cells due to limited acess to patient material. We thus took advantage of a recently established cell line that allows studying the function of trimeric FcεRI *in vitro*. This new cell line model is based on MelJuso cells, which were stably transfected with FcεRI-alpha and FcεRI-gamma cDNA. The resulting MelJuso-αγ cells express FcεRI-alpha at the cell surface (Figure 3A) and can bind monomeric IgE (Figure 3B, left FACS histogram). In line with previous reports, IgE binding to MelJuso-αγ also induces upregulation of surface FcεRI-alpha, a key feature of this FC receptor (Figure 3B, right FACS histogram). Multimeric FcεRI complexes containing FcεRI-alpha and FcεRI-gamma subunits can be precipitated from this cell line (Figure 3C) and receptor activation by crosslinking of FcεRI induces efficient receptor internalization from the cell surface (Figure 3D). All of these features match the characteristics of trimeric human FcεRI found in the literature [10], [11], [12]. Thus, we used this cell model to address whether IgE-mediated activation of cell-surface FcεRI induces the release of the soluble form of the receptor. MelJuso-αγ cells were loaded overnight with hapten-specific cIgE. After removal of excess cIgE, surface FcεRI was activated by crosslinking the receptor-bound ligand with haptenized antigen. 36 h after receptor crosslinking, sFcεRI was precipitated from culture supernatants with a cIgE column and visualized by immunoblotting with mAb 19-1 and compared to sFcεRI precipitated from patient serum (Figure 3E). Next, the kinetics of sFcεRI release was studied by harvesting supernatants 4, 8, 24 and 32 h after receptor crosslinking for analysis by ELISA. Accumulation of sFcεRI was observed only after receptor crosslinking (Figure 3F, left graph). sFcεRI was not detected in supernatants of empty vector-transfected MelJusoØØ cells that do not express FcεRI (Figure 3F, right graph). To demonstrate that the detected protein was a soluble form of FcεRI and not protein shedded with exosomes or derived from cell debris, sequential high-speed ultracentrifugation was performed to deplete the supernatants from cell debris and exosomes as established by Thery *et al.* [30]. sFcεRI was detected in the exosome-depleted, soluble fraction after high-speed centrifugation confirming that the detected protein is a bona fide soluble form of the receptor (Figure 3G).

### sFcεRI inhibits IgE loading of FcεRI at the cell surface *in vitro*

**Figure 4:**
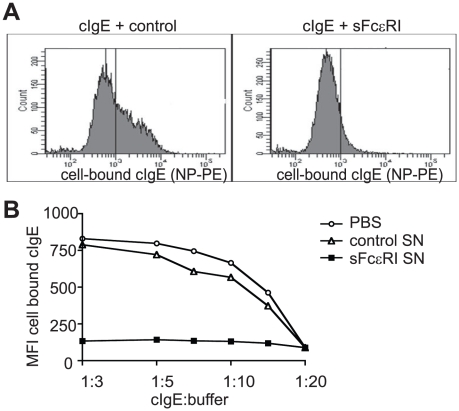
sFcεRI blocks IgE loading of cell surface-expressed FcεRI. A. MelJuso-αγ were loaded for 30 min on ice with a 1∶2 dilution of cIgE in PBS (left panel) or cIgE in cell culture supernatant containing sFcεRI (right panel). B. Serial dilutions of cIgE in a cell culture supernatant containing sFcεRI (black squares) or cIgE in supernatants derived from unstimulated MelJuso-αγ that did not contain sFcεRI (open triangles; control, open circles). Cell-bound cIgE was stained with PE-conjugated hapten and measured by flow cytometry. sFcεRI prevents cIgE binding to cellular FcεRI. MFI: mean fluorescence intensity.

Since we detected IgE-sFcεRI complexes in serum, we speculated that sFcεRI could interfere with IgE-binding to FcεRI when expressed at the cell surface. If that indeed occurs, sFcεRI could function as a potential modulator of IgE-mediated immune activation. We tested this hypothesis by loading FcεRI-expressing MelJuso-αγ cells with either a mix of cIgE and cell-culture derived sFcεRI or with cIgE diluted with medium control. Cell-bound cIgE was visualized by flow cytometry with PE-conjugated hapten NP. sFcεRI efficiently blocked binding of cIgE to FcεRI expressed at the cell surface (Figure 4A). Binding of cIgE was blocked in a dose dependent manner as dilution curves with supernatants from sFcεRI-containing MelJuso-αγ cells and control supernatants from inactivated cells demonstrated (Figure 4B). In summary, these results show that sFcεRI can interfere with binding of IgE to FcεRI at the cell surface of immune cells.

## Discussion

We here describe a soluble version of the FcεRI-alpha (sFcεRI) chain that circulates in human serum as a free protein or bound to its natural ligand, IgE. We show that sFcεRI is released upon IgE-antigen-mediated activation of cell surface FcεRI *in vitro* and, maybe most interestingly, that sFcεRI interferes with IgE-binding to cellular FcεRI *in vitro*. The affinity of IgE with its high affinity receptor FcεRI was defined after crystallization of the ligand with recombinant version of the alpha chain [31], [32]. It is therefore highly likely that the soluble form in human serum has equally high affinity as described in the literature.

Commonly, the reagents used to detect transmembrane forms of FcεRI-alpha are directed against the IgE-binding epitope of the protein. Thus, the identification of sFcεRI could easily have been missed if the detection reagents were not selected carefully. We here established an ELISA system that uses a monoclonal antibody directed against the stalk region of the protein [29] to capture sFcεRI and use human IgE combined with anti-IgE for detection [12]. By omitting the IgE incubation step, this ELISA also allows for an assessment of the amount of sFcεRI that circulates as a preformed complex with serum IgE.

Several studies with recombinant versions of sFcεRI are found in the literature [33], [34]. Since the recombinant sFcεRI used as a tool to interfere with allergic responses and a potential therapeutic agent, there have been some speculations about a soluble serum equivalent in the literature [8]. A single report is found in the literature that described a soluble complex of FcεRI in cultures of human eosinophils [35]. Since the integrity of FcεRI complexes requires the presence of cell membranes [28], [36], Seminario *et al.* most likely described a version of the receptor that was released in an exosomal fraction rather than a bona fide soluble protein.

Based on our current understanding of the mechanism of sFcεRI generation, it is fair to assume that serum sFcεRI is a reflection of FcεRI activation. In an independent study, we were able to confirm the observation of Liang *et al.* [37] showing that patients can carry substantial amounts of IgE on peripheral blood cells even in the absence of elevated serum IgE [27]. In summary, these two studies show that cells in the peripheral blood bind IgE from the serum and thereby can clear the serum of IgE. These IgE-loaded cells could be the source of sFcεRI when activated. Our finding that the presence of sFcεRI correlates with serum IgE supports this hypothesis. On the other hand, IgE-mediated cell activation could also account for the detection of serum sFcεRI in the absence of high serum IgE levels. Whether patients that have high sFcεRI are protected from allergic diseases will have to be addressed in detail. Along this line of argument, it is tempting to speculate that serum sFcεRI is a predictive marker for the onset of allergies that may be detectable even before serum IgE levels are elevated. We are currently investigating this hypothesis in a prospective cohort study.

sFcεRI is also an excellent candidate for an efficient *in vivo* modulator of IgE-mediated responses. While sCD23 has to trimerize to develop considerable affinity for its ligand [1], sFcεRI can bind IgE with a one-to-one ligand-receptor ratio. Additionally, the affinity of the FcεRI-IgE interaction is exceptionally high and disruption of a once formed contact requires low pH, which is physiologically found only in the stomach [1], [6], [7]. The finding that receptor crosslinking is required for the production of sFcεRI also hints at a potential negative feedback mechanism. Antigen-IgE-mediated receptor crosslinking could induce shedding of sFcεRI to remove IgE-binding sites from the cell surface and to terminate receptor-mediated signaling. In addition, we show here that sFcεRI has the ability to prevent IgE-binding to surface expressed receptors. Thus the presence of serum sFcεRI could inhibit IgE-loading of effector cells of allergy *in vivo*.

Our *in vitro* studies suggest that sFcεRI may also prevent IgE-mediated activation of the immune system by clearing the serum of IgE in a manner comparable to omalizumab. Omalizumab is a recombinant humanized monoclonal antibody directed against serum IgE and currently approved for the treatment of severe allergic asthma [38], [39], [40]. Omalizumab also downregulates cell surface levels of FcεRI [41].

In summary we here describe a new soluble form of human FcεRI, the high affinity receptor for IgE (s FcεRI), in human serum. Establishing an improved quantitative ELISA with a standard protein is now highly important, because such a quantitative method will allow for an extensive comparative analysis of serum levels in various patient groups. Such studies will be of outmost importance to draw conclusions about the clinical relevance of this new serum IgE receptor. It will be also essential to gain a better understanding of how endogenous levels of sFcεRI are regulated and how sFcεRI is linked to IgE-mediated immune activation *in vivo*.

## Materials and Methods

### Ethics statement

Patient sera used for this study were obtained from an ongoing prospective cohort study on the role of FcεRI in the gastrointestinal tract at Children's Hospital Boston or had been previously obtained as part of routine clinical care at the Medical University of Vienna. The prospective cohort study was approved by the Investigational Review Board of Children's Hospital Boston (Harvard Medical School, Boston, MA) and patients or their legal guardians provided written informed consent. The retrospective study of patient sera was approved by the Ethics Committee of Medical University of Vienna, Vienna, Austria.

### Antibodies and reagents

Anti-human FcεRI alpha mAb 19-1 was kindly provided by Dr. J.P. Kinet (Laboratory of Allergy and Immunology, Beth Israel Deaconess Medical Center, Boston, MA) and used as previously described [12], [28], [36]. Anti-human FcεRI alpha mAb CRA1 (clone AER-37) was purchased from eBioscience, San Diego, CA. Anti-FcεRI-gamma polyclonal serum was purchased from Millipore, Billerica, MA. Chimeric IgE that contains the human Fc domain and recognizes the haptens 4-hydroxy-3-nitrophenylacetic acid (NP) and 4-hydroxy-3-iodo-5-nitrophenylacetic acid (NIP) with its Fab region (cIgE) was derived from Jw 8/5/13 cells (Serotec, Oxford, UK, kindly provided by Dr. D. Maurer, Department of Dermatology, Medical University of Vienna, Austria, [12], [42]) and was used for immunoprecipitation of properly folded FcεRI-alpha and for *in vitro* cell culture experiments. Phycoerythrin (PE)-conjugated was purchased from Biosearch Technologies, Novato, CA, and used for flow cytometry analysis. Anti-mouse IgG (Fc specific, produced in goat; Sigma Aldrich, St. Louis, MO, #M3534-1 mL) was used for coating of the ELISA plates. High-IgE human serum (total IgE>2000 kU/L) was purchased from Bioreclamation, Hicksville, NY to assure the quality control of IgE used for detection of sFcεRI by ELISA. Goat anti-human IgE HRP conjugated antibody (Caltag, Invitrogen, Carlsbad, CA) was used as a secondary antibody.

### Cell lines and culture conditions

The MelJuso cell line was provided by Hidde L. Ploegh (Whitehead Institute, MIT, Cambridge, MA) and is well-established for studies on MHC class I and MHC class II trafficking [43]. MelJuso cells that stably express FcεRI-alpha and FcεRI-gamma (MelJuso-αγ) were generated by viral transduction using a standard protocol (http://www.stanford.edu/group/nolan/) following the guidelines of Children's Hospital Boston. MelJuso cells transfected with empty vector (MelJusoØØ) and MelJuso-αγ cells were maintained in Dulbecco's Modified Eagle Medium (DMEM, Cellgro, MediaTech, Herndon, VA) supplemented with 10% fetal calf serum (HyClone, Logan, UT), 2 mM glutamine (Cellgro), 100 U/ml penicillin, and 100 µg/ml streptomycin (Gibco BRL, Gaithersburg, MD). Cells were reselected using hygromycin (1 mg/ml) and puromycin (1 µg/ml, both Sigma). Jw 8/5/13 cells [12], [42] were used for the production of cIgE and cultured in RPMI 1640 medium (Gibco, Invitrogen, Grand Island, NY) supplemented with 10% fetal calf serum (HyClone), 2 mM glutamine (Cellgro), 100 U/ml penicillin, and 100 µg/ml streptomycin (Gibco BRL).

### Patient sera

Sera from adult individuals were tested for the presence of sFcεRI by immunoblot and ELISA. Sera from eight poly-sensitized, highly atopic children were analyzed for sFcεRI by ELISA. Total serum IgE and allergen-specific IgE were measured by solid phase immunoassay (Phadia ImmunoCAP®, Pharmacia Diagnostics, Uppsala, Sweden). Total serum IgE levels are given in kU/l, specific IgE is given in kUA/l and CAP RAST classes.

Sera from 119 children were obtained from an ongoing prospective cohort study on the role of FcεRI in the gastrointestinal tract. Patients between 1 and 19 years of age scheduled for an elective esophago-gastro-duodenoscopy at the Division of Gastroenterology at Children's Hospital Boston were randomly invited to participate. Subjects who used steroids in any form, immunomodulatory drugs, mast cell stabilizer, or leukotriene inhibitor within the last 3 months, as well as patients with an established diagnosis of autoimmune, inflammatory, or immunodeficiency disease were not enrolled. Total serum IgE levels were assessed according to standard procedures at Children's Hospital Boston using the Elecsys IgE II kit (Roche Diagnostics, Mannheim, Germany). IgE levels are given in kU/l. Expected normal ranges for this assay are 30 kU/l for age 0–3 years, 200 kU/l for 3–10 years, 500 kU/l for 10–14 years, and 200 kU/l for >14 years.

### Immunoprecipitation and immunoblotting of sFcεRI

To target the fully mature form of FcεRI alpha as expressed on the cell surface, we loaded MelJuso-αγ cells with cIgE (10 mg/ml in PBS) before solubilization in lysis buffer (3×10^6^ cells per ml; 0.5% Brij 96, 20 mM Tris, pH 8.2, 20 mM NaCl, 2 mM EDTA, 0.1% NaN_3_) containing protease inhibitors (Complete, Roche, Genentech, South San Francisco, CA) for 30 min on ice. Immunoprecipitation was next performed with NIP-beads (Sigma) as previously described [36], [42]. Proteins were eluted from beads in non-reducing Laemmli sample buffer and samples were separated on 12% non-reducing SDS-PAGE gels, transferred to PVDF membrane (Pierce, Thermo Fisher Scientific, Rockford, IL) and probed with anti-FcεRI-alpha (mAb 19-1 or CRA1 for reducing conditions, both 0.5 mg/ml) followed by peroxidase (HRP)-conjugated goat-anti-mouse IgG for detection of precipitated α-chain. For immunoprecipitation of sFcεRI from serum, 2–5 ml serum was used. cIgE-loaded NIP columns were also used for purification of sFcεRI from supernatants of MelJuso-αγ cells prior to immunoblotting. Peroxidase activity was detected by SuperSignal chemiluminescent substrate (Pierce). Accordingly, sFcεRI was precipitated from serum with IgE-coupled beads and immunoblotting was performed.

For co-immunoprecipitation of FcεRI alpha and gamma chains from MelJuso-αγ, cells were loaded overnight with cIgE. Cell lysates were prepared and incubated with NIP sepharose beads as described above. FcεRI alpha-chain was detected with mAb 19-1, FcεRI gamma-chains with polyclonal rabbit anti-FcεRIγ antibodies (Upstate).

### ELISA for the detection of sFcεRI in cell culture supernatants and serum

To improve sensitivity, wells were first incubated with a goat-anti-mouse coating antibody (5 mg/ml, Sigma), then with anti-alpha chain mAb (CRA1 0.5 mg/ml, clone AER-37; eBioscience). After a blocking step with 10% FCS in PBS, wells were incubated with sera (1∶2 dilution) overnight. After repetitive washing, plate-bound sFcεRI was loaded with IgE (Bioreclamation) and detected with a goat anti-human IgE-HRP conjugated second step (Caltag). Rather than using IgE purified from different patient sera, IgE was purchased to ensure the quality and consistency of this reagent in our ELISA. Conversion of 3,3′,5,5′-tetramethyl-benzidine liquid substrate (TMB, Sigma) was measured at 450 nm. Results are given as optical density (OD). To control for intra-assay variability, we included on each plate a positive and a negative control sample consisting of a pool of three positive or three negative patients respectively. The levels of circulating IgE-sFcεRI complexes were determined by omitting the IgE-loading step of the protocol. Levels of free sFcεRI were then calculated as follows: OD_total sFcεRI_−OD_IgE- sFcεRI complexes_ = OD_free sFcεRI_.

In a subset of patients, sFcεRI was measured in plasma and serum in parallel. For conversion of plasma samples into serum, BD Serum Separation Tubes (Becton Dickinson) were used according to the manufacturer's guidelines.

### Production of sFcεRI by MelJuso-αγ cells and detection in cell culture supernatants

MelJuso-αγ cells were grown to confluence and incubated with cIgE overnight. Excess cIgE was washed away and ligand-bound receptor was activated with haptenized antigen (BSA- or OVA-, 1 µg/ml, both from Biosearch Technologies, Novato, CA). Cell culture supernatants were collected after the indicated time periods and analyzed for the presence of soluble alpha chain by ELISA or by immunoprecipitation.

### Exosome removal

To remove exosomes from cell culture supernatants, MelJuso-αγ supernatants were treated with a sequence of ultracentrifugation steps following the protocol published by Thery *et al.* [30]. Briefly, exosome-free supernatants were obtained by the following consecutive centrifugations: 300 g for 5 minutes, 1200 g for 20 min, and 10000 g for 30 min, followed by a final centrifugation step at 110000 g for 1 h.

### Flow cytometry analysis

Surface expression of FcεRI on MelJuso-αγ cells was determined by staining with the anti-human FcεRI alpha mAb CRA1. IgE binding was tested by culturing MelJuso-αγ cells in the presence or absence of NP-specific cIgE and staining with phycoerythrin (PE)-conjugated NP (NP-PE; Biosearch Technologies). For the detection of sFcεRI, cells were loaded with either a mix of cIgE (100 ng/ml) in culture supernatants that contained sFcεRI or supernatants from unstimulated cell cultures for 30 min on ice. A number of different ratios of cIgE to culture supernatants was analyzed. FcεRI-bound cIgE was stained with NP-PE. Analysis was performed on a BD FACScan™ flow cytometer using CellQuest software for acquisition and data analysis (both from Becton Dickinson).

### Immunofluoresces Microscopy

For FcεRI alpha internalization, MelJuso-αγ cells were grown on coverslips (No 1.5), stained first with purified mouse anti-FcεRI alpha CRA1 antibody for 20 min at 37°C and subsequently with an anti-mouse Alexa Fluor 568 for 45 min at 37°C to induce receptor crosslinking. Cells were fixed with 4% paraformaldehyde for 20 min and mounted using Prolong Antifade reagent (Invitrogen). Both antibodies were diluted in HBSS supplemented with 10 mM HEPES (Invitrogen) and 5% NuSerum (Invitrogen). CRA1 was diluted at 1∶100 and anti-mouse Alexa Fluor 568 was diluted at 1∶400. Plasma membranes of fixed cells were stained with Alexa Fluor 647-conjugated wheat germ agglutinin (WGA, diluted at 1∶1000) for 10 min. For time point t = 0 cells were fixed before incubation with anti-mouse Alexa Fluor 568 and WGA. All incubation steps were carried out in a humidified chamber. Confocal images were acquired on a Nikon TE2000 inverted microscope coupled to a Yokogawa spinning disk confocal unit (Perkin-Elmer Inc.) and an Orca AG scientific-grade cooled CCD camera (Hamamatsu Photonics K.K.). Slidebook software (Intelligent Imaging Innovations Inc.) was used for image capture, processing, and analysis.

### Statistical Analysis

Correlations between serum IgE and serum sFcεRI were calculated by Spearman's rank correlation test using SPSS for Windows (version 16.0, SPSS Inc., Chicago, IL). Spearman's rank correlation coefficients are displayed as ‘rho’, a p-value of >0.05 was considered significant.

## Supporting Information

Table S1 Serum levels of sFcεRI in atopic patients.
(DOC)
