# Supplementary material for: A Soluble Form of the High Affinity IgE Receptor, Fc-Epsilon-RI, Circulates in Human Serum
Source: PLoS One. 2011 Apr 22;6(4):e19098. doi: 10.1371/journal.pone.0019098 (PMC3081330; doi:10.1371/journal.pone.0019098)
Supplement: Table S1 — Serum levels of sFcεRI in atopic patients. (DOC) [file pone.0019098.s001.doc]

| **Pat. ID** | **Gender** | **Age (years)** | **Total IgE (kU/L)** | **Specific IgE (kUA/L, CAP RAST class)** | **sFcεRI (OD)** |
| --- | --- | --- | --- | --- | --- |
| 160 | f | 12.17 | 1318 | Timothy grass (61.1, class 5), rye (39.9, class 4), birch pollen (>100, class 6), ragweed (69.9, class 5), mugwort (0.91, class 2), cat (5.69, class 3), dog (1.24, class 2), cod (6.72, class 6), tuna (2.73, class 2), salmon (7.04, class 3), peanut (10.1, class 3), hazelnut (3.55, class 3), paranut (9.83, class 3), almond (0.71, class 2), orange (1.43, class 2), coconut (1.73, class 2), apple (26.7, class 4), banana (0.70, class 2), peach (5.12, class 3), latex (0.75, class 2) | 0.57 |
| 63 | f | 8.42 | 136 | Ragweed (15.1, class 3), peanut (22.0, class 4) | 0.44 |
| 70 | m | 11.75 | 298 | Cat (3.99, class 3), milk (3.37, class 2), peanut (4.09, class 3), soy bean (1.92, class 2), chicken (1.06, class 2) | 0.59 |
| 368 | m | 9.70 | 733 | Birch pollen (>100, class 6), *Der p 2* (1.13, class 2), dog (1.79, class 2), peanut (2.57, class 2), hazelnut ( >100, class 6) | 0.46 |
| 278 | f | 8.50 | 471 | Cat (6.69, class 3), dog (0.81, class 2), peanut (>100, class 6), soy bean (13.5, class 3), hazelnut (0.51, class 1), paranut (1.31, class 2) | 0.67 |
| 463 | m | 14.7 | 104 | Timothy grass (17.5, class 4), rye (15.3, class 3), birch pollen (3.77, class 3), mugwort (1.02, class 2), *Der p 2* (1.16, class 2), cat (4.52, class 3), egg (0.43, class 1), peanut (2.92, class 2), latex (1.97, class 2) | 0.3 |
| 235 | m | 6.08 | 2465 | Timothy grass (19.4, class 4), rye (20.3, class 4), birch pollen (>100, class 6), mugwort (8.85, class 3), cladosporium herbarum (6.85, class 3), *Der p 2* (5.7, class 3), cat (0.67, class 1), dog (1.69, class 2), egg (9.07, class 3), milk (1.11, class 2), fish (4.05, class 3), mussels (3.97, class 3), tuna (4.28, class 3), salmon (6.64, class 3), shrimp (31.2, class 4), wheat (7.79, class 3), peanut (>100, class 6), soy bean (88.0, class 5), hazelnut (>100, class 6), paranut (14.8, class 4), almond (11.7, class 3), coconut (8.7, class 3) | 3.06 |
| 201 | m | 11.25 | 997 | Birch pollen (>100, class 6), *Der p 1* (0.53, class 2), cat (0.88, class 2), dog (0.88, class 2), egg (0.80, class 2), milk (4.08, class 3), wheat (4.28, class 3), rye (4.84, class 3), peanut (6.22, class 3), soy bean (6.32, class 3), hazelnut (>100, class 6), paranut (3.87, class 3), almond (5.24, class 3), coconut (2.86, class 2) | 0.92 |

**Table SI. Serum levels of sFcεRI in atopic patients.**
